# E2F8 promotes hepatic steatosis through FABP3 expression in diet-induced obesity in zebrafish

**DOI:** 10.1186/s12986-015-0012-7

**Published:** 2015-05-20

**Authors:** Yasuhito Shimada, Shisei Kuninaga, Michiko Ariyoshi, Beibei Zhang, Yasuhiko Shiina, Yoshinori Takahashi, Noriko Umemoto, Yuhei Nishimura, Hiroyuki Enari, Toshio Tanaka

**Affiliations:** Department of Molecular and Cellular Pharmacology, Pharmacogenomics and Pharmacoinformatics, Mie University Graduate School of Medicine, 2-174, Edobashi, Tsu, Mie Japan; Department of Systems Pharmacology, Mie University Graduate School of Medicine, Mie, Japan; Mie University Medical Zebrafish Research Center, Mie, Japan; Department of Bioinformatics, Mie University Life Science Research Center, Mie, Japan; Department of Omics Medicine, Mie University Industrial Technology Innovation, Mie, Japan; Central Research Institute, Maruha Nichiro Corporation, Ibaraki, Japan

**Keywords:** Fatty liver, Obesity, Zebrafish, High-fat feeding, DNA microarray, Two-dimensional electrophoresis, Cross-species analysis

## Abstract

**Background:**

Diet-induced hepatic steatosis is highly associated with nonalcoholic fatty liver disease, which is related to the development of metabolic syndrome. While advanced stage nonalcoholic hepatic steatosis and steatohepatitis (NASH) result ultimately in fibrosis and cirrhosis, the molecular basis for lipid droplet formation is poorly understood. Common pathways underlie the pathology of mammalian obesity and the zebrafish diet-induced obesity model (DIO-zebrafish) used in this study.

**Methods:**

Our analysis involved a combination of transcriptome (DNA microarray) and proteome (two-dimensional electrophoresis) methods using liver tissue from DIO-zebrafish to find candidate genes involved in hepatic steatosis. We conducted intraperitoneal injection (i.p.) of morpholino antisense oligonucleotides (MOs) for each gene into DIO-zebrafish. We also conducted *in vitro* overexpression in human cells. Additionally, we examined gene expression during feeding experiments involving anti-obesity compounds, creatine and anserine.

**Results:**

We found that fatty acid binding protein 3 (fabp3) and E2F transcription factors were upregulated in hepatic steatosis. E2f8 MO i.p. suppressed *fabp3* expression in liver, and ameliorated hepatic steatosis. In human cells (HepG2), *E2F8* overexpression promoted *FABP3* expression. Additionally, co-administration of creatine and anserine suppressed obesity associated phenotypes including hepatic steatosis as indicated by *e2f8* and *fabp3* down regulation.

**Conclusion:**

We discovered that the e2f8–fabp3 axis is important in the promotion of hepatic steatosis in DIO-zebrafish. The combination of transcriptome and proteome analyses using the disease model zebrafish allow identification of novel pathways involved in human diseases.

**Electronic supplementary material:**

The online version of this article (doi:10.1186/s12986-015-0012-7) contains supplementary material, which is available to authorized users.

## Background

Hepatic steatosis is an accumulation of fat in the liver, and causes of non-alcoholic fatty liver disease (NAFLD) encompass a spectrum of diseases ranging from steatohepatitis (NASH) to increasing fibrosis and eventual cirrhosis [1], sometimes subsequent to hepatocellular carcinoma [2]. This is associated with metabolic syndrome, especially obesity, hyperlipidaemia and diabetes [3], and is now the most common liver disease in both adults and children worldwide [4]. The estimated prevalence of NAFLD in the general population ranges from 5 to 20 % and up to 75 % of patients with obesity and diabetes mellitus [5]. Elevated plasma concentrations of glucose and fatty acids promote hepatic fatty acid synthesis and/or impair β-oxidation leading to hepatic steatosis [6]. Lipid accumulation in the liver is also linked with the progression of endoplasmic reticulum and mitochondria stress, and impaired autophagy, resulting in the condition known as lipotoxicity [7]. Insulin resistance is one of the most important factors in the development of hepatic steatosis, however, pharmacological treatment related to insulin-sensitizing agents (metformin and thiazolidinediones [TZDs]) has been studied in hepatic steatosis with conflicting results in clinical situations [8]. This suggests that the primary molecular basis for formation of hepatic steatosis is not related to insulin resistance. In fact, many genes implicated in hepatic steatosis are not related to insulin signalling, including FABPs. Dysregulated FABPs have been associated with a number of diseases, including obesity and NAFLD [9]. Preventing hepatic steatosis is critical for blocking the initial stages of NAFLD. Although the therapeutic mechanisms and gene targets involved in the accumulation of triglycerides (TG) in the liver and subsequent hepatocellular damage are not fully characterized, it is thought the process involves conventional lipid metabolism with insulin resistance.

The zebrafish (*Danio rerio*), a small vertebrate, has recently emerged as a genetically tractable model animal for human diseases [10]. Zebrafish have multiple advantages, such as a high degree of genetic conservation compared with mammals, their ease of genetic manipulation, and large clutch sizes making them amenable to high-throughput screening, including behaviour analysis [11]. In addition, lipid metabolism in zebrafish is similar to humans in that absorption occurs through the intestine with the aid of bile produced in the liver [12], transport of cholesterol is mediated by lipoproteins [13], and TG is stored in visceral, subcutaneous, and intramuscular adipocyte deposits [14]. In light of these similarities to mammals and the advantages of the system, the zebrafish model has been used in the field of lipid metabolism research for studies of lipid-related diseases, including dyslipidaemia with hepatic steatosis induced by diet-induced obesity (DIO) [15, 16], atherosclerosis-induced by high-cholesterol diet [17] and in a transgenic model of obesity [14]. In our previous studies, transcriptome analyses showed that the common pathways in hepatic steatosis of DIO-zebrafish were highly consistent with human obesity and rodent models of DIO [16]. We have also clarified the anti-hepatic steatosis mechanism of the bioactive molecule, eriocitrin [18], using the DIO-zebrafish.

In this study, we discovered that the fatty acid binding protein 3 (fabp3) gene was involved in hepatic steatosis by using DNA microarray and two-dimensional electrophoresis (2-DE) analyses of DIO-zebrafish, and the knockdown of e2f transcription factor 8 (*e2f8*) suppresses the development of hepatic steatosis via downregulation of *fabp3* in DIO-zebrafish.

## Methods

### Ethics statement

This study has been approved by the Ethics Committee of Mie University, and was performed according to Japanese animal welfare regulations outlined in the ‘Act on Welfare and Management of Animals’ (Ministry of Environment of Japan) and complied with international guidelines. After the experiments, the fish were sacrificed by an overdose of anaesthetic solution, tricaine methanesulfonate (500 mg/L; Sigma–Aldrich, St. Louis, MO, USA), in system water buffered with sodium bicarbonate (0.7 g/litter; Wako Pure Chemicals, Osaka, Japan).

### DIO-zebrafish experiments

The zebrafish *AB* wild type was supplied by the Zebrafish International Resource Center (University of Oregon, Eugene, OR) and maintained in our facility according to the established protocols [19]. To induce DIO, zebrafish were assigned into each dietary group with five fish per 1.7-L tank. From 3–4 months post fertilization (mpf), zebrafish in the overfeeding group were fed three times per day with Hikari Labo M-450 (HL450; Kyorin, Hyogo, Japan) containing beef tallow (HL450-BT; 7 % weight volume/fish weight/day) as high-fat (HF) diet. The normal feeding (NF) group was also fed one time per day with HL450 (3 % weight volume/fish weight/day). Each group contained 10 zebrafish. Every week we measured the body weight and calibrated the feeding volume daily. For HL450-BT, we prepared HL450 with 15 % total fat with beef tallow (Wako Pure Chemicals). Nutrition facts and the fatty acid compositions of HL450 and HL450-BT were described in Table 1 and Additional file 1: Table S1. Anserine (L-anserine nitrate salt; Sigma–Aldrich) and creatine (creatine anhydrous; Wako Pure Chemicals) were added to HL450-BT (1.34 mg/gBW/day and 0.14 mg/gBW/day, respectively), according to our previous method [20]. Zebrafish were fed to satiation three times daily. Satiation was defined as the point within a 5 min, where zebrafish were no longer actively searching for food [21]. Food consumption was calculated as the difference between the weight of food offered and food remaining.

**Table 1:** Nutrition information of fish foods

Nutrition information Per container (100 g)	HL450	HL450-BT
Energy (kcal)	379	405
Water (g)	7.4	7.5
Protein (g)	41.7	40.0
Lipid (g)	9.8	14.6
Carbohydrate (g)	31.0	28.4
Ash (g)	10.1	9.5

### Measurement of zebrafish body weight, length, BMI, plasma TG and fasting blood glucose

The body weight and length of zebrafish were measured weekly throughout the study as described previously [15]. The body mass index (BMI) was calculated by dividing the body weight (g) by the square of the body length (cm). For the blood chemistry analyses, zebrafish were deprived of food overnight and blood was withdrawn from the dorsal artery by a heparinized glass capillary needle (GD-1; Narishige, Tokyo, Japan) at the end of the feeding experiment. Fasting blood glucose and plasma TG were measured as described previously [22].

### Oil Red O staining

Liver tissues were collected from zebrafish by surgical manipulation under a MZ16F stereoscopic microscope (Leica Microsystems, Wetzlar, Germany). The preparation of liver sections and Oil Red O staining were performed as described previously [23]. Sections were also counterstained with Mayer’s hematoxylin (Wako Pure Chemicals, Osaka, Japan) to visualize the nuclei according to the manufacturer’s protocol. Lipid accumulation was quantified using the WinROOF version 5 (Mitani, Fukui, Japan).

### Measurement of liver lipid in zebrafish

Hepatic lipids were extracted from fixed liver tissues as described previously [24]. The fixation was conducted with RNAlater (Life Technologies) for at least 3-month at 4 °C. The dried lipid residues were dissolved in 15 μL of cyclohexane. Hepatic lipids were measured using the Lipid Quantification Kit (Colorimetric; Cell Biolabs, San Diego, CA, USA) according to the manufacturer’s protocol.

### Zebrafish DNA microarray

The samples for each condition were obtained from four independent experiments. DNA microarray experiments were conducted as described previously [16]. In brief, total RNA was extracted according to the protocol for Isogen (Nippon Gene, Tokyo, Japan), in combination with the clean-up protocol of the RNeasy Mini Kit (Qiagen, Hilden, Germany). The DNA microarray experiments were conducted using the Low RNA Input Fluorescent Linear Amplification Kit and G2518A Agilent Zebrafish Whole Genome Oligo Microarrays (Agilent Technologies, Santa Clara, CA, USA). The hybridized microarrays were scanned using an Agilent G2565BA microarray scanner and quantified using Feature Extraction software (Agilent Technologies). The data were further analysed using GeneSpring GX10 software (Agilent Technologies) to identify differentially expressed genes between two groups (*P* < 0.05). The probes were converted to human orthologs using the Life Science Knowledge Bank (World Fusion, Tokyo, Japan). Sub-network enrichment analysis (SNEA) [25] was conducted using Pathway Studio version 9 (Elsevier, Amsterdam, Netherlands).

### Zebrafish 2-DE

Liver tissues from three adult zebrafish were dissected and pooled in liquid nitrogen. The frozen tissues were then homogenized in ice-cold 85 % methanol. The homogenates were centrifuged at 12,000 × g for 20 min at 4 °C. The pellets were dissolved in lysis buffer (8 M urea, 2 M thiourea, 10 % isopropanol, 0.1 % Triton X-100, 4 % 3-[(3-cholamidopropyl) dimethylammonio]-1 propanesulfonate, and 50 mM dithiothreitol [DTT]), and were shaken for 40 min at room temperature. After precipitation (12,000 × g for 40 min at 20 °C), the protein concentration was estimated by Bradford assay (Bio-Rad, Richmond, CA, USA), and isoelectric focusing was performed on a 11-cm-long 3–10 nonlinear pI range IPG strip (Bio-Rad) using 200 μg of total liver protein (in 200 μL lysis buffer). Following a 4-h passive rehydration, isoelectric focusing was performed at 20 °C and 50 μA with the following four-step gradient program: 30 V for 10 h, 200 V for 1 h, 500 V for 1 h, 1000 V for 1 h, 8000 V for half an hour. The strips were then equilibrated in buffer I (6 M urea, 62.4 mM Tris [pH 6.8], 2 % sodium dodecyl sulphate, 4 % glycerol, 50 mM DTT, and 0.01 % bromophenol blue). Two-dimensional electrophoresis was performed in 12.5 % sodium dodecyl sulphate–polyacrylamide gel (11 × 11 cm) electrophoresis for 2 h at 40 mA. The gels were then fixed and stained (0.0028 % Coomassie Brilliant Blue R-250 and 28 % isopropanol) for 3 h and de-stained with 10 % acetic acid. The gels were scanned and documented using a GS-800 Calibrated Densitometer (Bio-Rad) and analysed using PD Quest 2-DE Analysis Software (Bio-Rad).

### Peptide mass fingerprinting

The protein band was cut and then transferred to a microtube loaded with 100 μl of 50 % acetonitrile containing Tris–HCl. Gel pieces were dehydrated twice with this 50 % ACN solution. The dried gel particles were rehydrated at 37 °C for 15 h using an XL-TrypKit (APRO Science, Tokushima, Japan) according to the manufacturer’s instruction. After trypsin digestion, the proteins were examined using a matrix-assisted laser desorption ionization time of flight mass spectrometer (Bruker Daltonics, MA, USA). The obtained peptide mass fingerprinting data were screened for preliminary protein IDs using GPS Explorer software version 3.6 (Applied Biosystems, Foster City, CA, USA) against the *Danio rerio* NCBI database (2008) and Swiss-Prot 2009 (MASCOT version 2.0; Matrix Science, Boston, MA, USA).

### Intraperitoneal administration of morpholinos

Morpholinos (MOs) were designed and synthesized by Gene Tools LLC (Philomath, OR, USA). The MO sequences are shown in Additional file 2: Table S2. For the negative control groups, the control MO (human β-globin mutant sequence; GeneTools) was used. Intraperitoneal (i.p.) administration of morpholinos was conducted as previously described [26]. In detail, 2 μl samples of each MO solution were diluted by adding 3 μl of OPTI-MEM I (Life Technologies), which was combined with a Lipofectamine 2000 (Life Technologies) mixture (2 μl of Lipofectamine 2000 and 3 μl of OPTI-MEM I). The MO mixtures were incubated at room temperature for 20 min and injected into the abdominal cavity of 3–4 mpf zebrafish (approximately 50 μmol/kg body weight) using FemtoJet (Eppendorf, Hamburg, Germany) with a fine-polished GD-1 glass capillary (Narishige, Tokyo, Japan). Intraperitoneal administration was conducted once a week during feeding experiments, starting from 1 week before the feeding experiment.

### Western blot

The liver tissues of DIO-zebrafish were collected by surgical extraction. Lysate protein was prepared by homogenization and sonication in T-PER Tissue Protein Extraction Reagent (Thermo Scientific, Rockford, IL) with protease inhibitor cocktail (Thermo Scientific). The samples were centrifuged at 12,000 × g for 30 min after homogenization with the MM300 Mixer Mill (30 Hz for 2 min; Retsch, Haan, Germany). For western blot analysis, protein samples were separated by 4–15 % SDS–PAGE and transferred onto a polyvinylidene fluoride (PVDF) membrane (Bio-Rad), and blocked at 20 °C with TBS containing 5 % skim milk (Becton, Dickinson and Company, Sparks, MD, USA) for 90 min. The membrane was incubated with goat polyclonal to E2F8 (1:2000; Aviva Systems Biology, San Diego, CA, USA) at 4 °C for 16 h, and then washed with TBS containing 0.05 % Tween-20 (TBST) five times. Horseradish peroxidase (HRP) -conjugated rabbit anti-goat (1:5000; Santa Cruz Biotechnology, Santa Cruz, CA, USA) secondary antibodies were used to detect E2F8. After five washes with PBST, immunoreactions were detected using TMB stabilized substrate for HRP (Promega, WI, USA) with a Molecular Imager Chemi Doc XRS Plus (Bio-Rad), and analysed using PD Quest Advanced/Basic Ver.8.0 (Bio-Rad).

### Cell culture and transfection

HepG2 human hepatocarcinoma cells were cultured in Dulbecco’s modified Eagle’s medium (Life Technologies, Carlsbad, CA, USA), supplemented with 100 μg/ml streptomycin sulphate (Sigma–Aldrich, St. Louis, MO, USA), 100 U/ml penicillin G (Sigma–Aldrich) and 10 % (v/v) foetal bovine serum (Life Technologies), and maintained at 37 °C in an atmosphere of 5 % CO_2_ and 95 % air. Cells were transfected at 70 % confluence with 2 μg plasmid DNA by using Lipofectamine 2000 reagent (Life Technologies). Plasmids containing human E2F3 (FHC12471) and E2F8 (FHC24174) were purchased from Promega (Madison, WI, USA). HaloTag Control Vector was used as a control for transfection. To confirm transfection efficacy, HaloTag TMR Ligand (Promega) was used according to the manufacturer’s protocol.

### qPCR

For liver tissues of adult zebrafish, total RNA of each sample was purified as described previously [27]. For cultured cells, total RNA was also purified using the RNeasy Mini Kit. First-strand cDNA was prepared with 200 ng total RNA using the Super Script III First-strand System (Life Technologies) with random primers (Life Technologies). qPCR was performed with Power SYBR Green Master Mix (Applied Biosystems) in triplicate, according to the manufacturer’s protocol. The sequences of the primers are shown in Additional file 3: Table S3. The oligonucleotides of these primers were synthesized by Life Technologies.

### Statistical analysis

All data were represented as mean ± SEM. Differences between the two groups were examined for statistical significance using Student’s *t* test. For multiple comparisons, we used one-way analysis of variance followed by Bonferroni–Dunn multiple comparison. *P* < 0.05 was considered to denote statistical significance.

## Results

### Proteome and transcriptome analyses of DIO-zebrafish

**Fig. 1:**
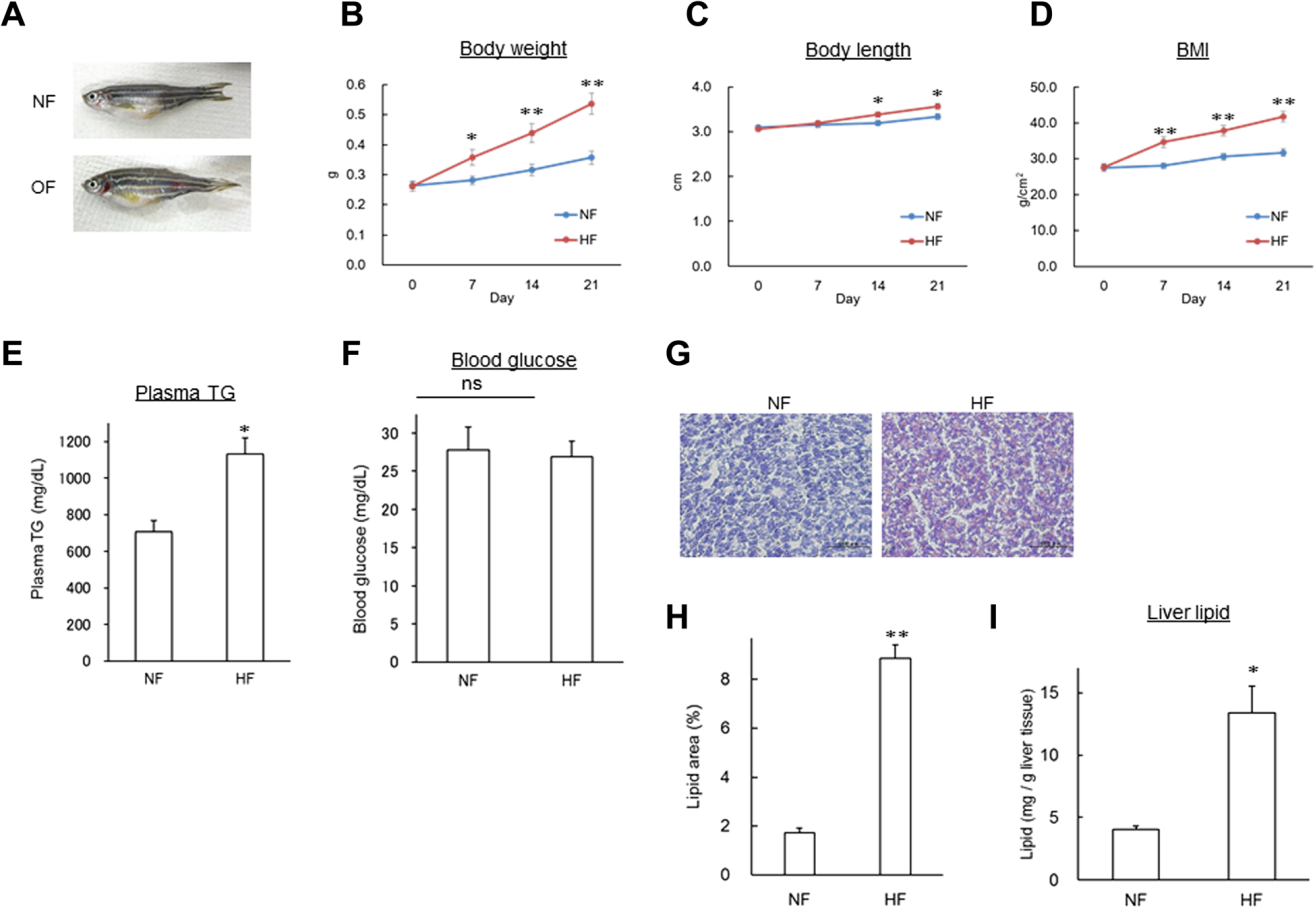
High-fat feeding-induced obesity with hepatic steatosis. (**a**) Typical images of NF and HF zebrafish. (**b – d**) Body weight (**b**), body length (**c**) and BMI (**d**) were increased by HF. n = 10, *P < 0.05, **P < 0.01. (**e – f**) HF increased plasma TG (**e**) on day 21, however it did not alter the level of fasting blood glucose (**f**). n = 10, *P < 0.05. ns means no significant difference. (**g**) Oil Red O staining of liver. Red colour indicates lipid droplets in the liver. (**h**) Quantification of the red area in (**g**). n = 10, **P < 0.01. (**i**) HF increased the level of hepatic lipids more than NF. n = 4, *P < 0.05

Typical image of DIO-zebrafish was shown in Fig. 1a. HF significantly (*P* < 0.05) increased body weight after 7 days of feeding (Fig. 1b). Body length was also increased by HF (Fig. 1c), while the increased rate was less than that for body weight (1.06-fold vs. 1.5-fold on day 21). Body mass index (BMI) was also significantly (*P* < 0.05) increased after 7-day feeding experiments (Fig. 1d). The plasma TG was increased significantly (*P* < 0.05, Fig. 1e) on day 21, however fasting glucose was not affected by HF (Fig. 1f). Lipid accumulation in liver tissues was also significantly (*P* < 0.05) increased in the HF group (Fig. 1g and h). In addition, HF significantly (*P* < 0.05) increased the contents of hepatic lipids more than NF (Fig. 1i).

**Fig. 2:**
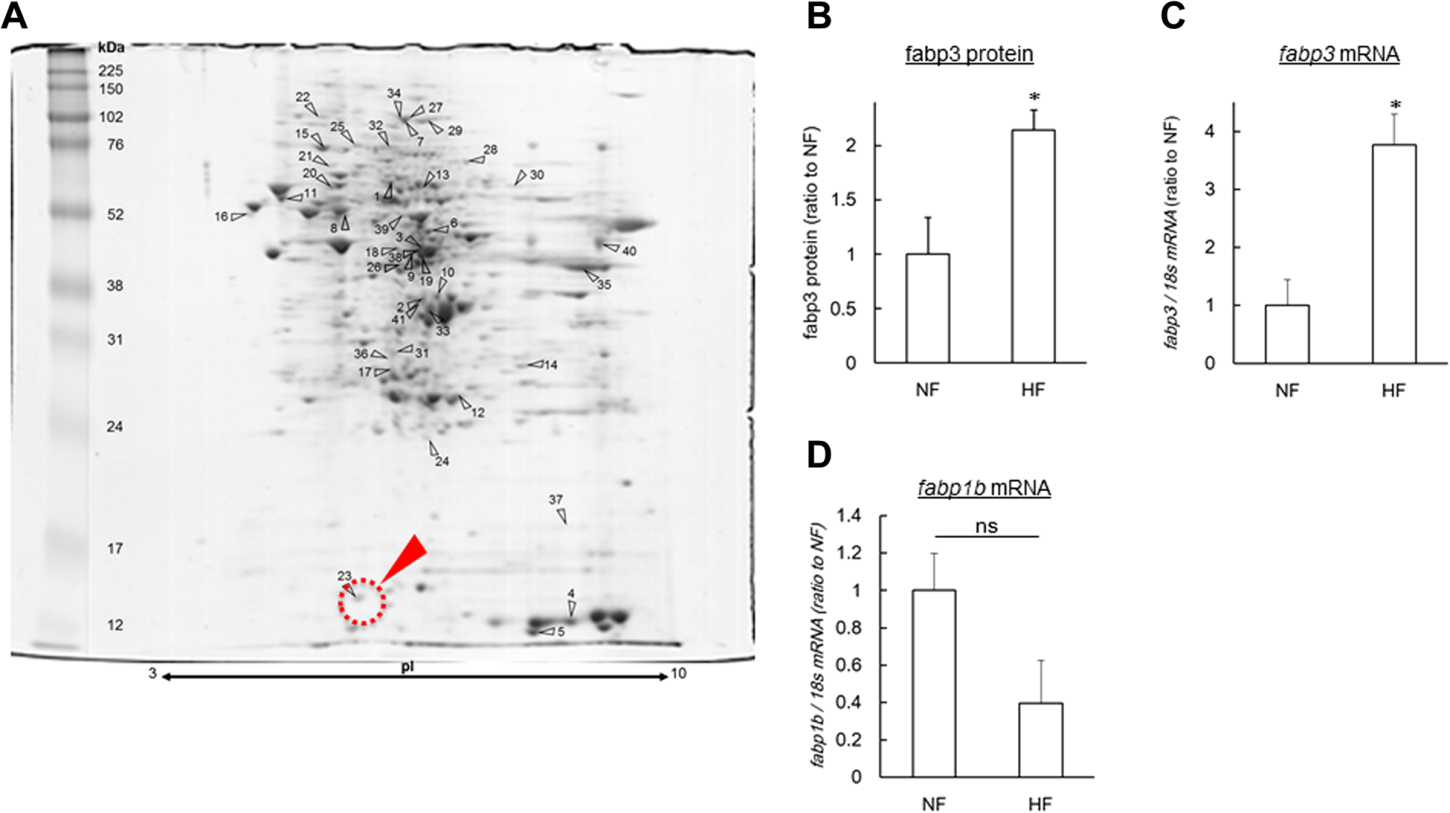
Fabp3 is increased in liver of DIO-zebrafish. (**a**) Gel image of 2-DE analysis of DIO-zebrafish liver. The spot numbers in the gel image correspond to Additional file 4: Table S4. Red arrow head indicates the spot of Fabp3 protein. (**b – c**) Fabp3 protein and *fabp3* mRNA expression were measured by 2-DE (**b**) and qPCR (**c**). n = 3–5, *P < 0.05. (**d**) *fabp1b* mRNA expression was measured by qPCR. n = 3–5

To identify the candidate gene products involved in the development of hepatic steatosis, we conducted proteome and transcriptome analyses of these liver tissues. For the 2-DE experiments, we detected 41 spots that were significantly (*P* < 0.05) altered in the HF group (Fig. 2a and Additional file 4: Table S4). These zebrafish proteins corresponded to 23 human orthologs. Analysis of the genes with altered expression by gene ontology category using GOStat [28] revealed that amino acid metabolism, glucose metabolism, endoplasmic reticulum and mitochondria functions were altered in the HF group (Additional file 5: Table S5).

**Fig. 3:**
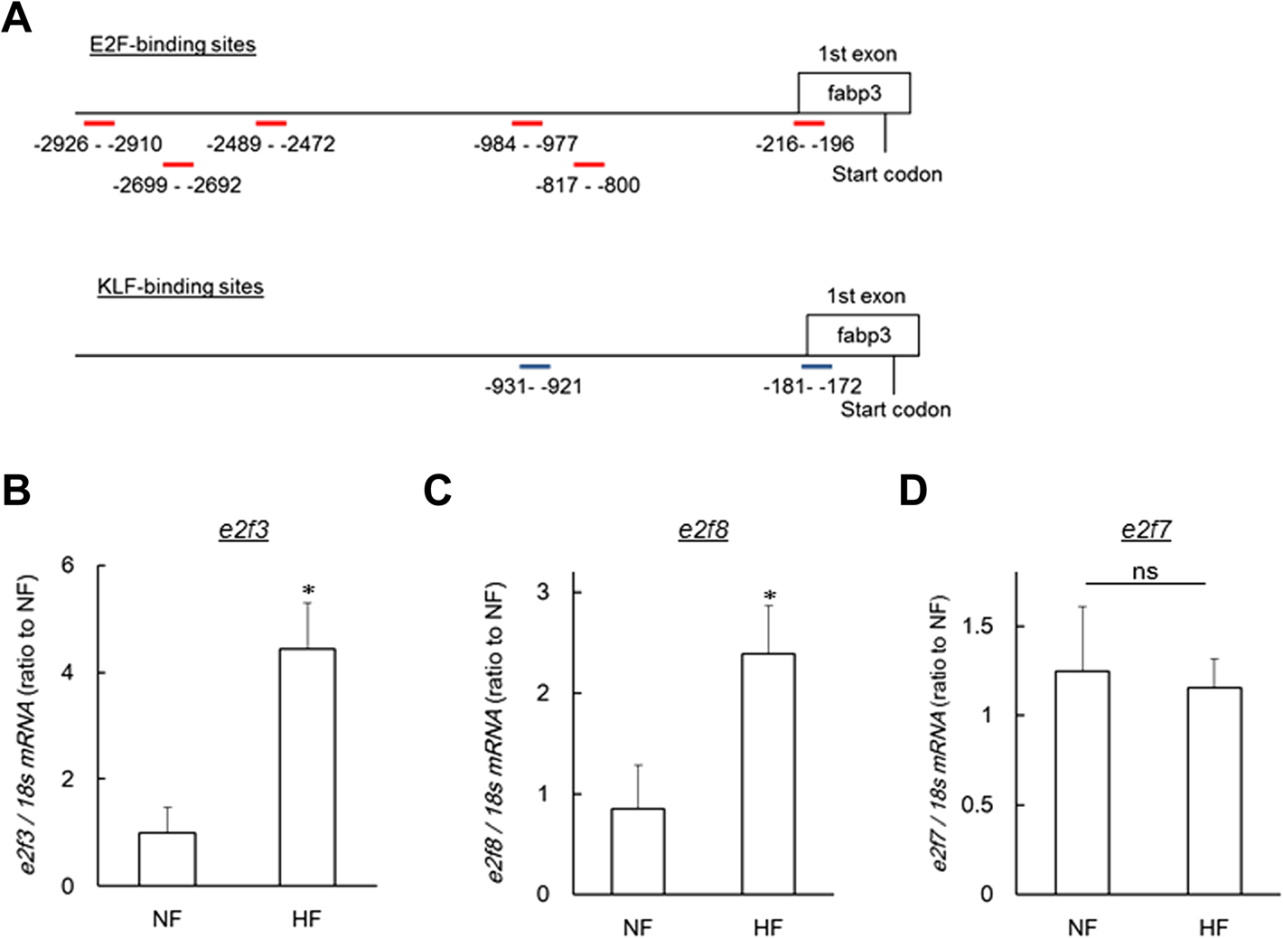
E2f3 and e2f8 are candidates of fabp3 transactivation in DIO-zebrafish. (**a**) E2F and KLF-binding site analysis of the zebrafish *fabp3* promoter region. Red and blue short bars represent binding sites of E2F and KLF, respectively. Numbers indicate the 5′ location of the binding site from the start codon of each gene. (**b-d**) E2f family gene expression in DIO-zebrafish. HF increased *e2f3* (**b**) and *e2f8* (**c**) expression, not *e2f7* (**d**). n = 4, *P < 0.05

Since the 2-DE analysis is limited to the genomic coverage, next we conducted transcriptome studies using DNA microarrays. We found that 125 and 63 human orthologs are significantly (*P* < 0.01) increased and decreased by HF, respectively (Additional file 6: Table S6). Table 2 shows the list of altered genes common to 2-DE and DNA microarray. Of these, fatty acid-binding protein 3 (fabp3) protein and mRNA were significantly (*P* < 0.05) increased in HF (Fig. 2b and c). For the liver-specific fabp isoform, *fabp1b*, there is no significant difference between NF and HF groups (Fig. 2d). Next, we conducted sub-network enrichment analysis (SNEA) [25] with DNA microarray data, which predicts the upstream pathways from the list of altered genes (Additional file 7: Table S7). In this list, we predicted two groups of transcription factors, E2F transcription factors and Kruppel-like factor (KLF) transcription factors, would be involved in the development of the hepatic steatosis. Next, we conducted *in silico* promoter analysis of zebrafish *fabp3* using the JASPAR CORE database [29]. We found that the *fabp3* promoter has six E2F-binding sites and two KLF-binding sites, respectively (Fig. 3a). Of the E2F family genes, we found that *e2f3* and *e2f8* were significantly (*P* < 0.05) increased in the HF group by qPCR (Fig. 3b and c). The expression change of *e2f3* is consistent with the DNA microarray result. In DNA microarray analysis, *e2f4* was also decreased in the HF group (Additional file 6: Table S6), however expression of *e2f4* was not detected by qPCR. *E2f7* expression was significantly (*P* < 0.01) increased in the DNA microarray experiment (Additional file 6: Table S6), but there was no significant difference between the NF and HF groups (Fig. 3d).

**Table 2:** Gene list common to 2-DE and DNA microarray

Protein name	ZF Gene ID	Human ortholog	Human Gene ID	2-DE	DNA microarray
heat shock 70 kDa protein 8	573376	HSPA8	3312	2.66	0.64
valosin containing protein	327197	VCP	7415	2.25	1.50
fatty acid-binding protein, heart (FABP3)	171478	FABP3	2170	2.15	2.49
Adenosylhomocysteinase	387530	AHCY	191	0.49	0.66
haemoglobin subunit alpha	30507	HBZ	3050	0.21	0.52

### Fabp3, e2f3 and E2f8 knockdown suppressed lipid accumulation of liver in DIO-zebrafish

**Fig. 4:**
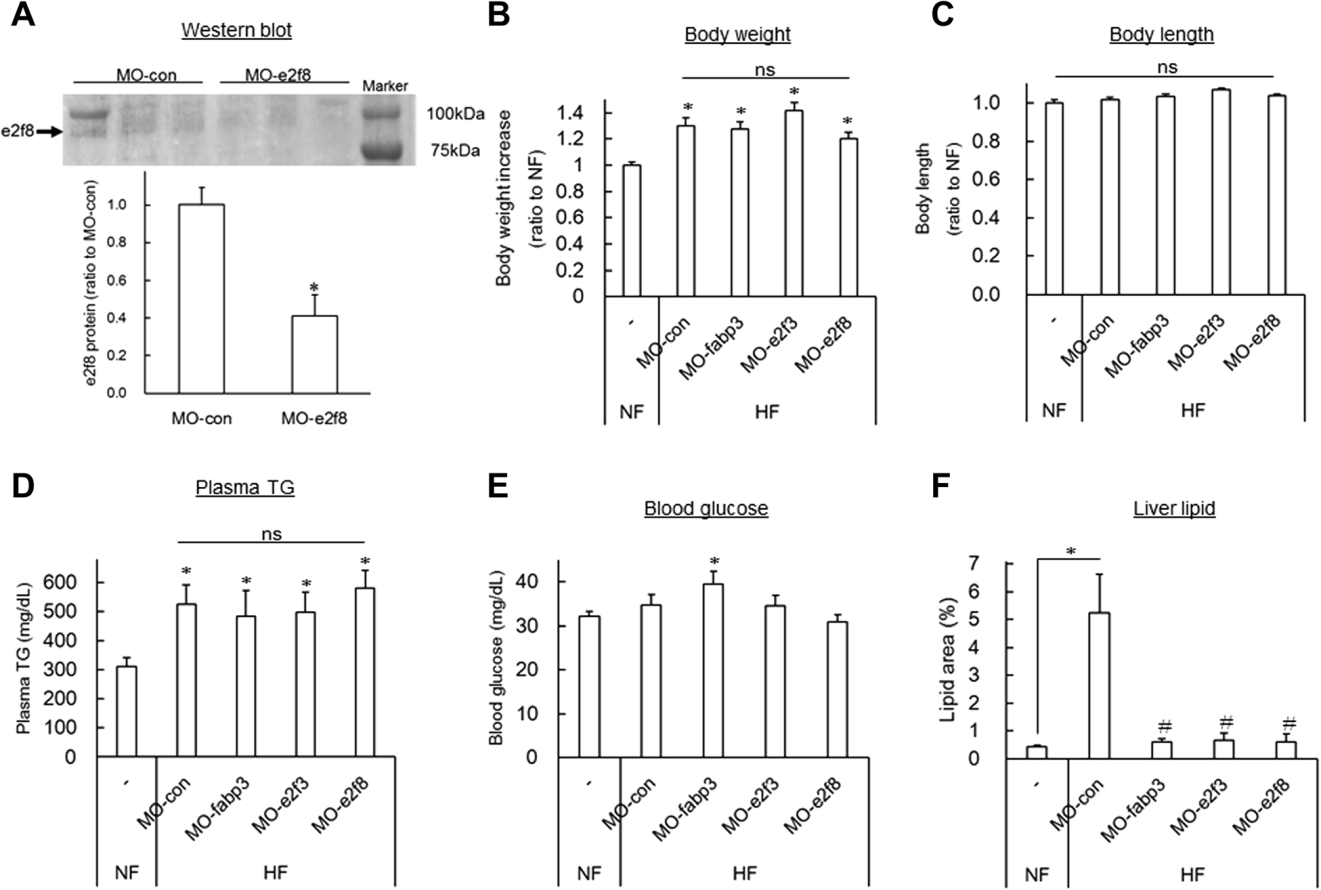
Effects on fabp3, e2f3 and e2f8 morpholino in DIO-zebrafish. (**a**) E2f8 Western blot of MO-e2f8 and control MO administration in HF. n = 3, *P < 0.05. (**b-c**) MO i.p. administration did not affect body weight (**a**) and body length (**b**) in DIO-zebrafish on day 14. MO-con indicates control MO as a negative control. n = 5, *P < 0.05 vs. NF. (**d**) MO i.p. administration did not affect plasma TG in DIO-zebrafish on day 14. n = 5, *P < 0.05 vs. NF. (**e**) MO-fabp3 i.p. administration increased the level of fasting blood glucose, while HF + control MO (MO-con) did not. n = 5, *P < 0.05 vs. NF. (**f**) The area of lipid droplets of Oil Red O staining of liver. All MOs suppressed liver steatosis in DIO-zebrafish. n = 5, *P < 0.05 vs. NF, #*P* < 0.05 vs. HF+ MO-con

To examine the contribution of e2f3, e2f8 and fabp3 to the development of hepatic steatosis, we conducted fabp3, e2f3 and e2f8-specific MO (MO-fabp3, MO-e2f3 and MO-e2f8) i.p. administration every week during feeding experiments of DIO-zebrafish as previously described [26]. There was no appetite suppression by MOs during feeding experiments (data not shown). On day 14, after MO-e2f8 administration three times (starting from 1 week before the feeding experiment), e2f8 protein was reduced to almost 40 % that of control MO with HF (Fig. 4a). We also tried e2f3 and fabp3 Western blot, but could not succeeded, probably because of the difference of immunogen’s sequence between mammals and zebrafish. There was no difference in body weight (Fig. 4b) and length (Fig. 4c) between these i.p.-administered MOs in DIO-zebrafish. Plasma TG was not affected by these MOs (Fig. 4d), however fasting blood glucose was increased by MO-fabp3 (Fig. 4e). All of these MOs (fabp3, e2f3 and e2f8) reduced lipid accumulation of liver tissues more than HF with control MO, which was detected by Oil Red staining (Fig. 4f).

### E2F8 regulates FABP3 expression in zebrafish and HepG2 cells

**Fig. 5:**
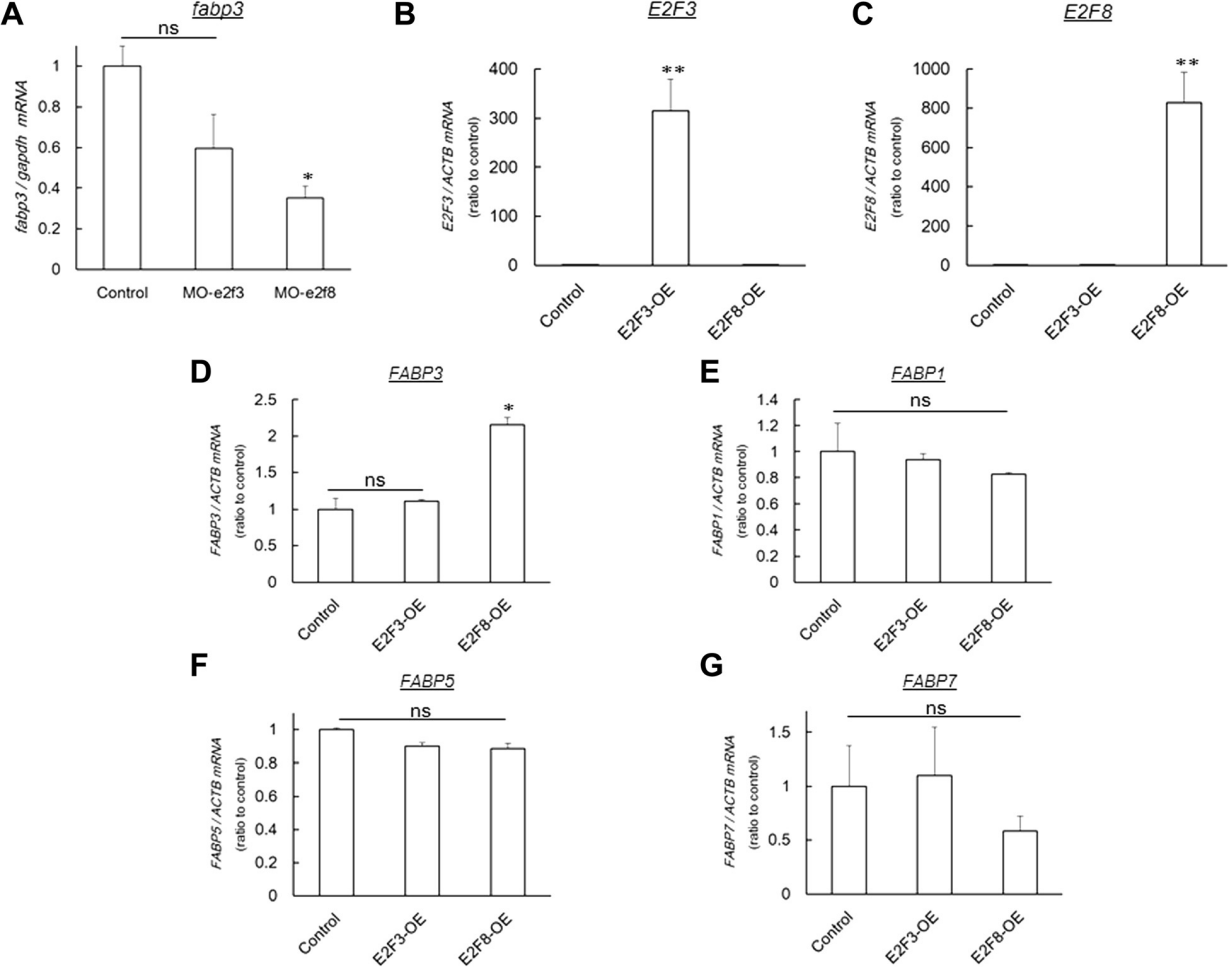
E2f8 regulates *fabp3* expression in zebrafish and human cells. (**a**) qPCR analysis of *fabp3* in zebrafish analysed in Fig. 4. *Fabp3* expression was suppressed by MO-e2f8 i.p. n = 5, *P < 0.05 vs. control MO (MO-con). (**b-c**) qPCR analysis of forced expression of *E2F3* (**b**) and *E2F8* (**c**) in HepG2 cells. n = 4, **P < 0.01 vs. control. (**d-g**) FABP family gene expression in E2F3-or E2F8-overexpressed (OE) cells. FABP3 were induced only in E2F8-OE cells (**d**), while FABP1 (**e**), FABP5 (**f**) and FABP7 (**g**) were not altered in these OE cell lines. n = 4, *P < 0.05 vs. control

In the DIO-zebrafish, MO-e2f8 significantly (*P* < 0.05) suppressed *fabp3* mRNA expression in liver (Fig. 5a). The MO-e2f3 showed a trend to suppress *fabp3* expression without significance. To examine whether the alterations of *fabp3* expression by these MO-i.p. could also be extrapolated to humans, we conducted forced expression of *E2F3* and *E2F8* in HepG2 human hepatocarcinoma cells (Fig. 5b and c). *E2F8*, not *E2F3*, overexpression increased *FABP3* expression significantly (*P* < 0.05; Fig. 5d). Other FABP family, *FABP1*, *FABP5* and *FABP7* expression levels were not altered by *E2F3* or *E2F8* overexpression (Fig. 5e-g). These results indicates that E2F8 could positively regulate *FABP3* expression in human and zebrafish.

### Co-administration of anserine and creatine suppressed obesity-associated phenotypes via downregulation of fabp3

**Fig. 6:**
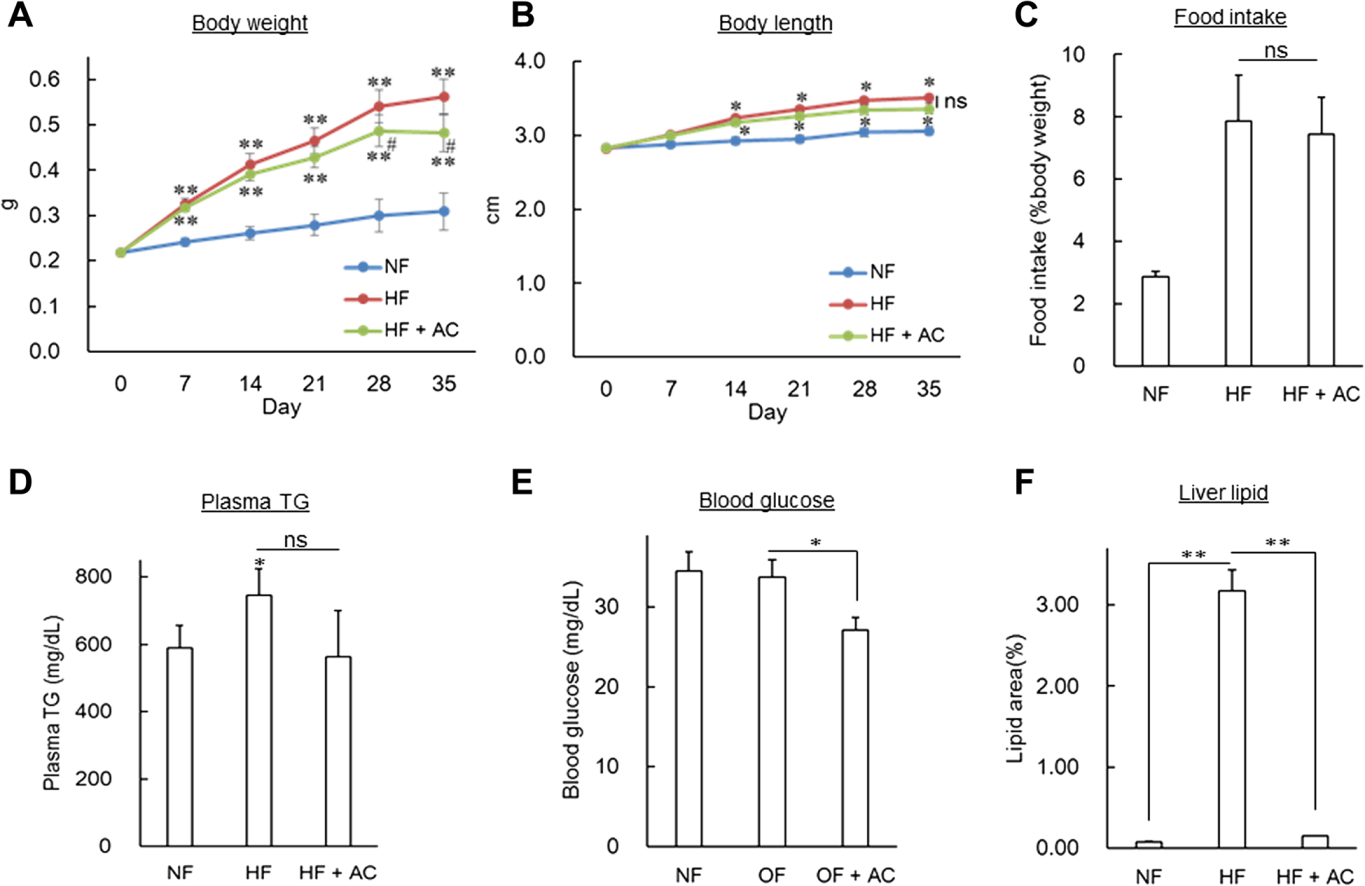
Anserine and creatine feeding to DIO-zebrafish. (**a – b**) Body weight and body length in the feeding experiment. Co-administration of anserine and creatine (AC) suppressed body weight increase (**a**), but not body length (**b**). n = 8, *P < 0.05 and **P < 0.01 vs. NF, and #*P* < 0.05 vs. HF. (**c**) Food intake during the feeding experiment. There is no difference between HF and HF + AC. n = 8. (**d**) For plasma TG, there is no difference between HF and HF + AC. n = 5-6, *P < 0.05 vs. NF. (**e**) AC decreased the level of fasting blood glucose. n = 4-6, *P < 0.05. (**f**) The area of lipid droplets of Oil Red O staining of liver. AC suppressed lipid accumulation in the liver of DIO-zebrafish. n = 7-8, **P < 0.01

**Fig. 7:**
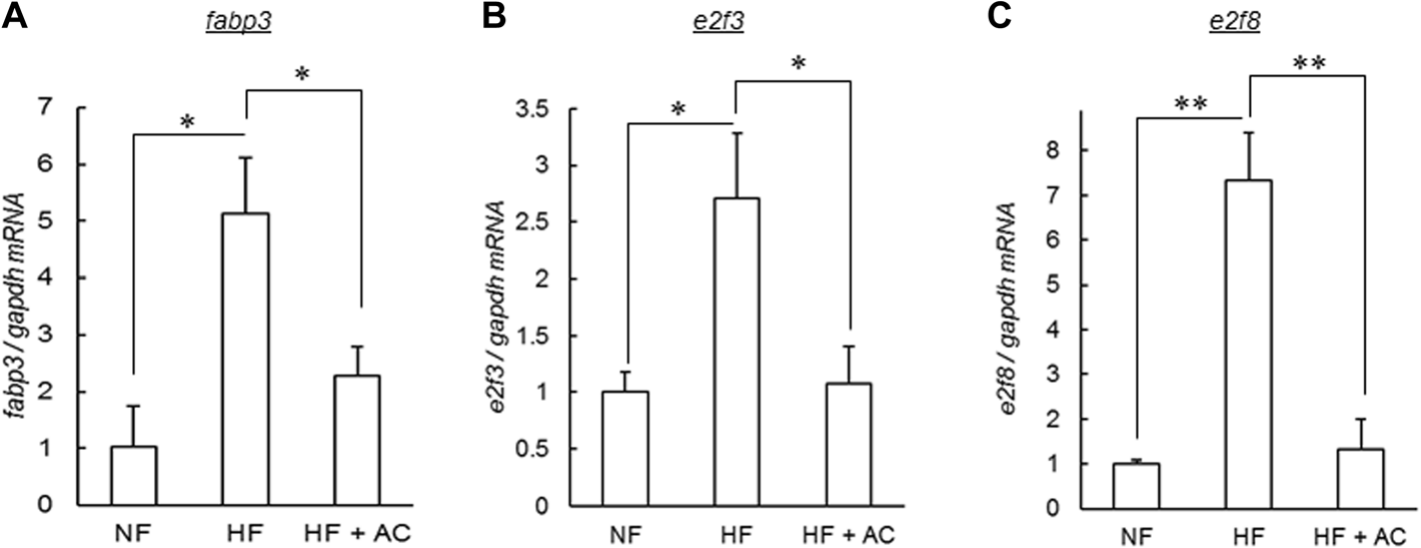
Anserine and creatine effects on *fabp3*, *e2f3* and *e2f8* expression. (**a-c**) qPCR analysis of AC-administration in DIO-zebrafish. *Fabp3* (**a**), *e2f3* (**b**) and *e2f8* mRNA (**c**) were increased by HF, and then decreased by AC (HF + AC group). n = 4, *P < 0.05, **P < 0.01

To confirm that *fabp3* expression is responsible for the improvement of hepatic steatosis, we administered anserine and creatine to DIO-zebrafish as anti-obesity therapeutics. Anserine and creatine are known to alter insulin secretion and glycogen metabolism, changes that ameliorate the obesity phenotype in a rodent model of obesity [30, 31] and in human clinical trials [32]. We fed anserine and creatine-containing food (AC) to DIO-zebrafish for 21 days. AC significantly (*P* < 0.05) suppressed the increase in body weight (Fig. 6a) without an effect on body length (Fig. 6b) or on feeding volume (Fig. 6c). Although AC could not suppress the increase in plasma TG (Fig. 6d), AC suppressed the increase in fasting blood glucose significantly (*P* < 0.05 vs. HF; Fig. 6e), similar to the previous study in rodent models [30, 31]. AC also suppressed the accumulation of lipid droplets in liver tissues (*P* < 0.01, Fig. 6f). qPCR analysis of the liver tissues revealed that the increases in *fabp3*, *e2f8* and *fabp3* expression were also suppressed by AC administration (*P* < 0.05 vs. HF, Fig. 7a-c) in the liver.

## Discussion

### FABP3 involvement in hepatic steatosis

FABP family members are in charge of regulating fatty acid uptake and intracellular transport. Zebrafish have seven fabp family genes, which correspond to mammalian FABP1, FABP2, FABP3, FABP6, FABP7, FABP10 and FABP11. FABP3 regulates metabolic homeostasis and developmental regulation by modulating intracellular lipid transport in cardiac muscles of human [33] and zebrafish [34, 35]. FABP3 mRNA has been shown to be markedly up-regulated in liver and adipose tissue in rats with high-cholesterol diet [36, 37]. Furthermore, hesperidin, a citrus bioflavonoid, ameliorated liver steatosis in high-cholesterol diet rats through FABP3 down regulation in the liver [37]. In the Gene Expression Omnibus (GEO) [38], FABP3 was significantly (*P* < 0.05) increased in mouse livers with a ketogenic diet (GDS2738 [39]). The ketogenic diet is “high-fat, low carbohydrate”, similar to the HF group in our zebrafish experiments. In human, Başar O et al. reported increased human FABP3 concentrations in non-alcoholic fatty liver disease [40], metabolic disease [41] and in conditions of impaired glucose metabolism [42]. In zebrafish, *fabp3* is expressed in the liver of adult fish [43] and was induced by high-linoleic acid-rich foods [44]. In contrast to *fabp3*, FABP1, a liver-type FABP, is increased in hepatic steatosis in mammals and has a protective role against lipid toxicity [9]. The zebrafish homolog of human FABP1, fabp1b, is required for liver development [45]; however, the relationship between fabp1b and hepatic steatosis has not been reported. In our study, zebrafish fabp1b shows a decreasing trend in the HF group without significance (Fig. 2d), partially consistent with the down regulation in hepatic steatosis in mammals. Unlike mammals, it is noteworthy that fabp3 is expressed in liver, not in the heart of adult zebrafish [43], suggesting the possibility that fabp3 shares a function in lipid metabolism with fabp1b.

### *The E2F8*–*FABP3 pathway promotes hepatic steatosis*

In the current study, SNEA and promoter analysis predicted the involvement of the E2F family in hepatic steatosis. E2F family members play a major role during the G1/S transition of the cell cycle across phyla from plants to mammals. The E2F family is generally split by function into two groups: transcriptional activators (E2F1, E2F2 and E2F3a) and transcriptional repressors (E2F3b, E2F4, E2F5, E2F6, E2F7 and E2F8) [46]. However, recent studies of mouse knockout tissues demonstrate that E2F function *in vivo* does not strictly adhere to this elegant dichotomous paradigm [47]. E2Fs also regulate the expression of the metabolic genes involved in hepatic steatosis [48] and obesity [49]. There is a limited number of studies related to E2F8 and obesity, however, these are still controversial. E2F8 expression was increased (about 7-fold) in the adipose tissue of DIO-mice [50], similar to our result. Partial deficiency in the retinoblastoma protein gene, which is upstream of E2F8 [51], protects against the development of obesity and associated metabolic disturbances [52], also consistent with our results. Peroxisome proliferator-activated receptor β (PPARβ) knockout (KO) suppressed E2F8 mRNA expression in liver regeneration in mice [48]. Since activation of the PPARβ/δ complex inhibits hepatic steatosis [53], PPARβ KO-induced E2F8 suppression seems to worsen hepatic steatosis, which seems to be contrary to our result with MO-e2f8. However, with anti-obese compounds, AC suppressed hepatic steatosis, consistent with mammals, through suppression of *e2f3*, *e2f8* and *fabp3*. Additionally, anserine and creatine administration to DIO-zebrafish ameliorated the hepatic steatosis with down regulation of e2f8 and fabp3 in the current study. Creatine normalizes PPARα downregulation in HF-rats [54], and PPARα agonist induced FABP3 gene expression [55]. Thus we hypothesized that E2F8 transcription factor mediated FABP3 transactivation might be a downstream of PPARα in the development of hepatic steatosis. These results strengthened the notion that the E2F-FABP3 pathway is involved in hepatic steatosis. *E2f3* was also increased in the liver of DIO-zebrafish, and MO-E2F3 ameliorated the phenotype independent of *fabp3* expression. While no studies have examined the relationship between E2F3 and hepatic steatosis, E2F3 was found to be increased in the liver of HF-induced hepatic steatosis of mice (GDS4013 [56]) and clinical alcohol-induced hepatitis (GDS4389 [57]) in GEO. These results indicate that E2F3 would also be a strong candidate to promote hepatic steatosis. In fact, Asp P *et al.* found that mouse E2f3b, a shorter isoform of E2f3, bind a large number of lipid metabolism genes in myogenic differentiation [58], suggesting that E2F3 also might be involved in hepatic steatosis which is independent to FABP3. The actual DNA-binding affinity of E2F8 to FABP3 promoter region and its transactivation will be elucidated by ChIP analysis. Additionally, to confirm the common function of the E2F8-FABP3 pathway between zebrafish and mammals in hepatic steatosis, *in vitro* and mammalian model experiments should be completed in future.

## Conclusions

Using proteome and transcriptome analysis in DIO-zebrafish, we discovered that the E2F8-FABP3 pathway is one of the contributing factors to promoting hepatic steatosis in DIO-zebrafish. Combination analysis of the *in vivo*, *in vitro* and *in silico* data will hopefully lead to this pathway being a therapeutic target against diet-induced hepatic steatosis.

## Additional files

Additional file 1: Table S1. Fatty acid composition of fish foods. Additional file 2: Table S2. MO sequences. Additional file 3: Table S3. Primer sequences for qPCR. Additional file 4: Table S4. Protein list altered in DIO-zebrafish. Additional file 5: Table S5. Gene ontology analysis of proteins altered in DIO-zebrafish. Additional file 6: Table S6. Gene list altered in DIO-zebrafish. Additional file 7: Table S7. SNEA analysis.

## Abbreviations

2-DE: Two-dimensional electrophoresis
AC: Anserine and creatine-containing food
BMI: Body mass index
DIO-zebrafish: Diet-induced obesity zebrafish
DTT: Dithiothreitol
E2F: E2F transcription factor
FABP: Fatty acid binding protein
GEO: Gene expression omnibus
HL450: Hikari Labo M-450
HL450-BT: Hikari Labo M-450 containing beef tallow
i.p.: Intraperitoneal injection
KLF: Kruppel-like factor
MO: Morpholino antisense oligonucleotides
mpf: Months post fertilization
NAFLD: Non-alcoholic fatty liver disease
NASH: Non-alcoholic steatohepatitis
NF: Normal feeding
PPAR: Peroxisome proliferator-activated receptor
SNEA: Sub-network enrichment analysis
TG: Triacylglyceride
